# Seroprevalence of Antibodies to Severe Acute Respiratory Syndrome Coronavirus 2 Among Healthcare Workers in Kenya

**DOI:** 10.1093/cid/ciab346

**Published:** 2021-04-23

**Authors:** Anthony O Etyang, Ruth Lucinde, Henry Karanja, Catherine Kalu, Daisy Mugo, James Nyagwange, John Gitonga, James Tuju, Perpetual Wanjiku, Angela Karani, Shadrack Mutua, Hosea Maroko, Eddy Nzomo, Eric Maitha, Evanson Kamuri, Thuranira Kaugiria, Justus Weru, Lucy B Ochola, Nelson Kilimo, Sande Charo, Namdala Emukule, Wycliffe Moracha, David Mukabi, Rosemary Okuku, Monicah Ogutu, Barrack Angujo, Mark Otiende, Christian Bottomley, Edward Otieno, Leonard Ndwiga, Amek Nyaguara, Shirine Voller, Charles N Agoti, David James Nokes, Lynette Isabella Ochola-Oyier, Rashid Aman, Patrick Amoth, Mercy Mwangangi, Kadondi Kasera, Wangari Ng’ang’a, Ifedayo M O Adetifa, E Wangeci Kagucia, Katherine Gallagher, Sophie Uyoga, Benjamin Tsofa, Edwine Barasa, Philip Bejon, J Anthony G Scott, Ambrose Agweyu, George M Warimwe

**Affiliations:** 1 KEMRI–Wellcome Trust Research Programme, Kilifi, Kenya; 2 KEMRI Center for Infectious and Parasitic Diseases Control Research, Alupe, Kenya; 3 Kilifi County Hospital, Kilifi, Kenya; 4 Department of Health, Kilifi County, Kenya; 5 Kenyatta National Hospital, Nairobi, Kenya; 6 Alupe Sub-County Hospital, Busia, Kenya; 7 Institute of Primate Research, Nairobi, Kenya; 8 Kocholia Sub-County Hospital, Busia, Kenya; 9 Busia County Referral Hospital, Busia, Kenya; 10 Department of Health, Busia County, Busia, Kenya; 11 Department of Infectious Diseases Epidemiology, London School of Hygiene and Tropical Medicine, London, United Kingdom; 12 Ministry of Health, Government of Kenya, Nairobi, Kenya; 13 Presidential Policy and Strategy Unit, The Presidency, Government of Kenya, Nairobi, Kenya; 14 Nuffield Department of Medicine, Oxford University, Oxford, United Kingdom

**Keywords:** SARS-CoV-2, Healthcare Workers, Antibodies, Seroprevalence

## Abstract

**Background:**

Few studies have assessed the seroprevalence of antibodies against severe acute respiratory syndrome coronavirus 2 (SARS-CoV-2) among healthcare workers (HCWs) in Africa. We report findings from a survey among HCWs in 3 counties in Kenya.

**Methods:**

We recruited 684 HCWs from Kilifi (rural), Busia (rural), and Nairobi (urban) counties. The serosurvey was conducted between 30 July and 4 December 2020. We tested for immunoglobulin G antibodies to SARS-CoV-2 spike protein, using enzyme-linked immunosorbent assay. Assay sensitivity and specificity were 92.7 (95% CI, 87.9-96.1) and 99.0% (95% CI, 98.1-99.5), respectively. We adjusted prevalence estimates, using bayesian modeling to account for assay performance.

**Results:**

The crude overall seroprevalence was 19.7% (135 of 684). After adjustment for assay performance, seroprevalence was 20.8% (95% credible interval, 17.5%–24.4%). Seroprevalence varied significantly (*P* < .001) by site: 43.8% (95% credible interval, 35.8%–52.2%) in Nairobi, 12.6% (8.8%–17.1%) in Busia and 11.5% (7.2%–17.6%) in Kilifi. In a multivariable model controlling for age, sex, and site, professional cadre was not associated with differences in seroprevalence.

**Conclusion:**

These initial data demonstrate a high seroprevalence of antibodies to SARS-CoV-2 among HCWs in Kenya. There was significant variation in seroprevalence by region, but not by cadre.

## Teaser

In this study of antibodies to severe acute respiratory syndrome coronavirus 2 among healthcare workers in 3 counties in Kenya, we found significant regional differences in seroprevalence but no differences according to professional cadre.

Healthcare workers (HCWs) are critical in the acute-care response to epidemic waves of coronavirus disease 2019 (COVID-19), but they are also required to sustain normal health services beyond COVID-19. HCWs are considered to be at high risk of infection with severe acute respiratory syndrome coronavirus 2 (SARS-CoV-2) [1]. It is unclear whether the seroprevalence of SARS-CoV-2 antibodies among HCWs is more closely associated with community or hospital-based transmission risk, as indicated by professional cadre. In some hospitals, seroprevalence was higher among cadres in lower-paid jobs with little patient contact (eg, housekeepers and porters), suggesting that the source of infection may be their crowded living conditions rather than occupational risk [2]. The true extent of infection in HCWs in Kenya has been difficult to determine, owing to factors including the large proportion of asymptomatic infections (>90%) [3], possibly because of the young population structure, and challenges in the polymerase chain reaction testing of nasal and oropharyngeal swab samples in Kenya [4] and, indeed, most low- and middle-income countries.

Serological surveys can estimate cumulative incidence of SARS-CoV-2 infection either in key groups, such as HCWs, or in the general population [5]. They can also assess the effectiveness of infection prevention and control measures, which is important in sub-Saharan Africa where the availability of personal protective equipment and other preventive measures is constrained. To date, HCW serosurveys in sub-Saharan Africa have been limited to urban hospitals [6–8]; there are no surveys from rural hospitals, where resources are even more constrained. Serosurveys on different population groups or in different geographic regions can also inform vaccine prioritization policies. This is especially important in low- and middle-income countries, where only a small proportion of the population is likely to receive vaccines in the early phase of the vaccine campaign [9].

Because the presence of antibodies to SARS-CoV-2 appears to be strongly protective against repeated infection over a 6-month period [10, 11], knowledge of past infection could be useful for avoiding unnecessary quarantines, which would help preserve the limited numbers of personnel available to deal with the pandemic and other health needs in the region. We report initial findings from SARS-CoV-2 antibody testing from HCWs in 3 sites in Coastal, Central, and Western Kenya.

## METHODS

### Study Sites and Participants

Study sites (Supplementary Figure 1) were selected after consultation with the individual county COVID-19 rapid response teams. For Kilifi County, a predominantly rural area located on the Indian Ocean coast, we enrolled participants at Kilifi County Hospital, the main referral facility in the region. For Busia County, which is also predominantly rural and located in the western region of Kenya, we enrolled HCWs at Busia County Referral Hospital, the main referral facility in the area, and 2 other facilities in the county, Alupe Sub-County Hospital, designated as the isolation facility for patients with COVID-19 in the county, and Kocholia Sub-County Hospital. In Nairobi County, the capital city of Kenya, we enrolled HCWs at the Kenyatta National Hospital, the main referral facility for the city as well as the country [12].

We used a variety of strategies to recruit a convenience sample of HCWs at each of the study sites, including word of mouth, advertising at hospital notice boards, and messages sent via mobile phone. HCWs of all cadres were eligible to participate in the study. In Kilifi and Busia we aimed to recruit ≥50% (n = 441) of the 882 HCWs working in the healthcare facilities, which we considered to be both feasible and likely to provide a representative sample. We used a slightly different strategy at Kenyatta National Hospital, where the primary aim of the study was to determine incidence and antibody kinetics among approximately 180 HCWs, comprising approximately 4% of the hospital’s estimated 5000 HCWs [12], who were likely (by self-report) to be available for a year-long longitudinal study.

### Ethical Approval and Consents

Serosurveillance was conducted as a public health activity requested by the Kenyan Ministry of Health, and ethical approval for collection and publication of these data was obtained from the Kenya Medical Research Institute Scientific and Ethics Review Unit (KEMRI/SERU/CGMR-C/203/4085). HCWs provided written and/or verbal informed consent for participation in the study. Results of the antibody testing were reported confidentially to each HCW, together with information explaining the implications of the test results.

### Sample Collection and Processing

The study took place between 30 July and 4 December 2020. Data collection was performed by members of staff from the participating hospitals, trained on the study procedures.

We collected 6 mL of venous blood in sodium heparin tubes from each participant. Serum was obtained by centrifuging the samples at 450*g* for 5 minutes before storage at –80ºC. Samples were then transported in dry ice to the KEMRI–Wellcome Trust research laboratories in Kilifi for assays. A simple 1-page questionnaire (provided in the Supplementary Material) was administered to the HCWs either electronically or on paper, to collect data on demographic and clinical characteristics.

### Enzyme-Linked Immunosorbent Assay for SARS-CoV-2 Spike Protein

All samples were tested at the KEMRI–Wellcome Trust Research Programme laboratories in Kilifi for immunoglobulin G to SARS-CoV-2 whole spike protein using an adaptation of the Krammer enzyme-linked immunosorbent assay [13]. We assumed an assay sensitivity of 92.7% (95% confidence interval [CI], 87.9%–96.1%) and specificity of 99.0% (98.1%–99.5%), based on previously conducted validation studies [14]. Results were expressed as the ratio of test optical density (OD) to the OD of the plate negative control; samples with OD ratios >2 were considered positive for SARS-CoV-2 immunoglobulin G.

### Statistical Methods

Continuous variables were summarized as means and standard deviations if normally distributed and medians with interquartile ranges for nonnormally distributed variables. Categorical data were presented as counts and percentages. Bayesian modeling was used to adjust seroprevalence estimates for the sensitivity and specificity of the assay. Noninformative priors were used for all parameters, and the models were fitted using the RStan software package [15] (see Supplementary Material for code). We tested for associations between seroprevalence and professional cadre and site, respectively, using multivariable logistic regression. All analyses were conducted using Stata (version 15) and R (version 3.6.1) software

## RESULTS

We recruited 684 HCWs from Nairobi, Busia, and Kilifi (Supplementary Figure 2 and Table 1). As a proportion of total staff at the facilities, we recruited 70% of HCWs in Kilifi, 50% in Busia, and approximately 4% in Nairobi. The mean age (standard deviation) of the participants was 35 (11) years, and 54% were female. Sixteen (2%) of the HCWs reported having acute respiratory symptoms at the time of sample collection.

**Table 1.** Characteristics of Study Participants

	Participants by Site and Dates of Sample Collection, No. (%)			
Characteristic	All Sites: 30 Jul to 4 Dec (n= 684)	Kilifi: 13 Oct to 4 Dec (n = 200)	Nairobi: 30 Jul to 25 Aug (n = 183)	Busia: 19–23 Oct (n = 301)
Female sex	372 (54)	113 (57)	99 (54)	160 (53)
Age group, y^a^				
18–30	232 (34)	65 (33)	67 (37)	100 (33)
31–40	226 (33)	69 (35)	54 (30)	103 (34)
41–50	117 (17)	34 (17)	31 (17)	52 (17)
51–60	85 (13)	20 (10)	20 (11)	45 (15)
>60	17 (3)	8 (4)	9 (5)	0 (0)
PCR swab sample previously collected	250 (37)	31 (16)	77 (43)	142 (47)
Previous swab sample positive	5 (2)	1 (3)	0 (0)	4 (3)
Symptoms at sample collection	16 (2)	0 (0)	16 (9)	0 (0)
Chronic illness^b^	18 (3)	0 (0)	18 (10)	0 (0)
Work in COVID-19 unit^c^	50 (7)	0 (0)	0 (0)	50 (16)
Cadre				
Nurse	152 (22)	42 (21)	50 (27)	60 (20)
Physician	85 (12)	21 (11)	53 (29)	11 (4)
Clinical officer	79 (12)	48 (24)	4 (2)	27 (9)
Support staff^d^	117 (17)	33 (17)	21 (11)	75 (25)
Pharmacy	19 (3)	4 (2)	7 (4)	8 (3)
Laboratory	64 (9)	13 (7)	7 (4)	44 (15)
Other^e^	168 (25)	39 (20)	41 (22)	76 (29)

Abbreviations: COVID-19, coronavirus disease 2019; PCR, polymerase chain reaction.

^a^Age was missing for 7 individuals.

^b^Chronic illnesses included hypertension, diabetes, asthma, and human immunodeficiency virus infection.

^c^None of the healthcare workers in Nairobi or Kilifi worked in a COVID-19 unit.

^d^Support staff included kitchen staff, patient porters, security staff, and records clerks.

^e^Other staff included hospital administrators, supervisors, cashiers, and accountants.

Of the 684 HCWs, 135 (19.7%) were seropositive for antibodies to SARS-CoV-2 (Table 2). After adjustment for test performance characteristics, the seroprevalence was 20.8% (95% credible interval [CrI], 17.5%–24.4%). Adjusted seroprevalence among the different cadres ranged from 12.5% (95% CrI, 5.4%–21.8%) among clinical officers to 34.2% (23.7%–45.8%) among physicians. Seroprevalence was higher among HCWs in Nairobi (43.8%; 95% CrI, 35.8%–52.2%) than among those in Kilifi (11.9%; 7.2%–17.6%) or Busia (12.6%; 8.8%–17.1%).

**Table 2.** Seroprevalence of Antibodies to Severe Acute Respiratory Syndrome Coronavirus 2 by Participant Characteristics

	Participants, No.		Seroprevalence, %	
Characteristic	Total	Seropositive	Crude	Adjusted (95% CrI), %^a^
Age group, y				
18–30	232	49	21.1	22.1 (16.5–28.5)
31–40	226	46	20.4	21.6 (15.8–27.8)
41–50	117	20	17.1	18.3 (11.6–26.7)
51–60	85	15	17.7	18.8 (10.9–28.5)
>60	17	4	23.5	27.9 (9.3–50.8)
Sex				
Female	372	69	18.6	19.3 (15.1–24.1)
Male	312	66	21.2	22.1 (17.2–27.7)
Cadre				
Nurse	152	29	19.1	20.2 (13.8–27.9)
Physician	85	27	31.8	34.2 (23.7–45.8)
Clinical officer	79	9	11.3	12.5 (5.4–21.8)
Support staff	117	25	21.3	22.9 (15.2–31.6)
Pharmacy	19	5	26.3	30.3 (11.3–51.9)
Laboratory	64	12	18.8	20.4 (10.6–31.5)
Other	168	28	16.7	17.5 (11.8–24.3)
Site				
Kilifi	200	23	11.5	11.9 (7.2–17.6)
Nairobi	183	75	41.0	43.8 (35.8–52.2)
Busia	301	37	12.3	12.6 (8.8–17.1)
**Total**	**684**	**135**	**19.7**	**20.8 (17.5–24.4)**

Abbreviation: CrI, credible interval.

^a^Seroprevalence figures are adjusted for test performance; see Supplementary Material for code.

Table 3 displays the results of univariable and multivariable logistic regression modeling testing associations between participant characteristics and seroprevalence. The only exposure variable that displayed a statistically significant association with seroprevalence in the multivariable model was site; HCWs in Kilifi (OR, 0.2; 95% CI, .1–.3) or Busia (0.2; .1–.4) were less likely to be seropositive than those in Nairobi. Professional cadre, age, and sex were not associated with seroprevalence in either univariable and multivariable analyses. Site-specific analyses also did not reveal any association between seroprevalence and professional cadre (Supplementary Table 1).

**Table 3.** Univariable and Multivariable Analysis of Factors Associated With Presence of Antibodies to Severe Acute Respiratory Syndrome Coronavirus 2

	OR (95% CI)	
Characteristic	Univariable	Multivariable^a^
Sex		
Female	1.0	1.0
Male	1.18 (.81–1.71)	1.13 (.75–1.72)
Age (per decade)	1.00 (.98–1.01)	0.99 (.98–1.01)
Site		
Nairobi	1.0	…
Kilifi	0.19 (.11–.32)	0.18 (.10–.33)
Busia	0.20 (.13–.32)	0.21 (.13–.36)
Working in COVID-19 unit	0.33 (.12–.94)	0.51 (.17–1.55)
Symptoms at sample collection	1.98 (1.08–3.63)	1.34 (.78–2.30)
Chronic illness	2.49 (.88–6.97)	0.91 (.30–2.72)
Cadre		
Nurse	1.0	1.0
Physician	1.97 (1.07–3.63)	1.20 (.61–2.35)
Clinical officer	0.55 (.24–1.21)	0.97 (.41–2.30)
Support staff	1.15 (.63–2.09)	1.56 (.80–3.07)
Pharmacy	1.51 (.51–4.54)	1.50 (.45–4.97)
Laboratory	0.98 (.46–2.06)	1.45 (.64–3.27)
Other	0.85 (.48–1.50)	0.97 (.53–1.81)

Abbreviations: CI, confidence interval; COVID-19, coronavirus disease 2019; OR, odds ratio.

^a^Adjusted for all variables in table.

## DISCUSSION

We report results of a SARS-CoV-2 seroprevalence study conducted among HCWs in 3 counties in Kenya. We found an overall seroprevalence of SARS-CoV-2 antibodies of 20.8% (95% CrI, 17.5%–24.4%). There were significant differences in seroprevalence associated with hospital region, but no differences associated with professional cadre.

Our estimates of seroprevalence are higher than those in most of studies from Africa published to date, all of which were conducted in urban areas [6–8, 16] and had a pooled seroprevalence of 8.2% (95% CI, .8%–22.3%) [17]. We conducted our study during and shortly after the first wave of the epidemic in Kenya [4], while the previous studies in Africa were conducted relatively early in the epidemic. Our estimates are similar to those observed among HCWs in several high-income countries at the peak of their first wave of the epidemic [17].

Consistent with other studies conducted in Kenya [4, 14, 18–20], we found significant differences in seroprevalence by region. HCWs in urban Nairobi had significantly higher seroprevalence than HWCs in Busia and Kilifi, which are rural counties. Studies in Spain and India have also shown significant regional differences, with higher seroprevalence in urban areas, such as Madrid and New Delhi, compared with rural areas [21, 22]. However, even in the rural counties in Kenya, HCWs had seroprevalence estimates similar to those in HCWs in urban areas in Spain [23], the United States [24], and Malawi [6].

We found no differences in seroprevalence by professional category even when the analyses were stratified by study site. The absence of differences in seroprevalence by cadre in the presence of significant differences by geographic region suggests that community transmission could be playing a bigger role than workplace exposure. In studies of HCWs conducted in the United Kingdom, the incidence of infection mirrored that seen in the community [2, 25]. This suggests that efforts to suppress community transmission are likely to reduce infections among HCWs.

The results of the current study provide further evidence that there has been significant undocumented transmission of the SARS-CoV-2 virus within Kenya. Additional evidence of significant undocumented transmission in Kenya derives from (1) 2 studies of seroprevalence among blood transfusion donors [14, 18], (2) a study of truck drivers and their assistants conducted at the same time as this survey in Kilifi and Busia that found a seroprevalence of 42% [19], and (3) a study of antenatal clinic attendees, which found seroprevalence of 50% at Kenyatta National Hospital in August 2020 and 11% at Kilifi County Hospital in November 2020 [20].

A particular strength of this study is that we conducted it in several sites, which enabled us to detect a significant burden of infection among HCWs in rural parts of the country. Another strength is that we used an assay that was validated using both local and external samples and which performed well in a World Health Organization–sponsored international standardization study [26]. Although we adjusted our figures, using bayesian modeling to take into account assay performance, the reported seroprevalence could still be underestimated owing to antibody waning [27]. The longitudinal phase of the current study will help address this issue. Another possible reason for underestimation of the prevalence in our study would be spectrum bias [28], since though the samples that we used in validating the assay were derived from the local population, these individuals were not necessarily the same as the HCWs who participated in the present survey.

Our study had several limitations. We did not perform genetic sequencing to establish the likely sources of infections among the HCWs, although, as argued above, the data we obtained suggest that community transmission was the main driver of infections among the HCWs. The nonrandom selection of only a small proportion of the HCWs in Nairobi could have led to an overestimation of the seroprevalence if the HCWs sampled had an overrepresentation of individuals who had experienced symptoms in the past. However, this would have also resulted in a higher proportion of HCWs in Nairobi having positive results from previously conducted polymerase chain reaction tests, but we did not observe this. In addition a household survey found that 35% of the population in Nairobi had antibodies to SARS-CoV-2 [29], and the rural-urban difference in seroprevalence among HCWs that we observed was similar to what has been observed in other studies conducted in Kenya [14, 18–20].

In conclusion, we found a high prevalence of antibodies to SARS-CoV-2 among HCWs in Kenya, with significant regional differences and no differences based on cadre. The results suggest that infection with SARS-CoV-2 among HCWs is driven more by background population levels of infection than by workplace exposure and will be useful in informing measures to control the ongoing pandemic.

## Supplementary Data

Supplementary materials are available at Clinical Infectious Diseases online. Consisting of data provided by the authors to benefit the reader, the posted materials are not copyedited and are the sole responsibility of the authors, so questions or comments should be addressed to the corresponding author.

ciab346_suppl_Supplementary_Materials

## Notes

***Acknowledgments***. The authors thank all healthcare workers who participated in the study, as well as the county health teams and the Kenya Paediatric Research Consortium that facilitated data collection for the study. We thank F. Krammer for providing the plasmids used to generate the receptor-binding domain, spike protein, and CR3022 monoclonal antibody used in this work. The coronavirus disease 2019 (COVID-19) convalescent plasma panel (National Institute for Biological Standards and Control [NIBSC] 20/118) and research reagent for severe acute respiratory syndrome coronavirus 2 (SARS-CoV-2) antibodies (NIBSC 20/130) were obtained from the NIBSC, United Kingdom. They also thank the World Health Organization SOLIDARITY II network for sharing protocols and for facilitating the development and distribution of control reagents.

***Disclaimer.*** This manuscript was written with the permission of the director of KEMRI-Centre for Geographic Medicine-Coast (CGMRC). For the purpose of Open Access, the author has applied a CC-BY public copyright license to any author-accepted manuscript version arising from this submission.

***Financial support.*** This work was supported by the Wellcome Trust (grants 220991/Z/20/Z and 203077/Z/16/Z; Department for International Development (DFID)/Medical Research Council/National Institute for Health Research (NIHR)/Wellcome Trust joint global health trials award MR/R006083/1 to A. A; senior research fellowship 214320 to J. A. G. S.; grant 220985/Z/20/Z to C. N. A.; and intermediate fellowship 107568/Z/15/Z to L. I. O. O.); the Bill & Melinda Gates Foundation (grant INV-017547); the Foreign Commonwealth and Development Office through the East Africa Research Fund (grant EARF/ITT/039); the NIHR Health Protection Research Unit in Immunisation (J. A. G. S.); the United Kingdom’s Medical Research Council and Department for International Development (African research leader fellowship MR/S005293/1 to I. M. O. A.); the NIHR Mucosal Pathogens Research Unit at University College London (grant 2268427 through the London School of Hygiene and Tropical Medicine to I. A.); the Oak Foundation (G. M. W.); the DELTAS Africa Initiative (grants DEL-15-003 to C. N. A. and DEL-15-003 to S .U.); the Department for International Development (C. N. A.); and the National Institute of Allergy and Infectious Diseases Centers of Excellence for Influenza Research and Surveillance (contract HHSN272201400008C to support development of SARS-CoV-2 reagents. This work is part of an integrated program of SARS-CoV-2 serosurveillance in Kenya led by KEMRI–Wellcome Trust Research Programme.

***Potential conflicts of interest.*** J. A. G. S. reports grants from the Bill & Melinda Gates Foundation and Gavi, The Vaccine Alliance, outside the submitted work. All other authors report no potential conflicts.
