# Supplementary material for: Seroprevalence of Antibodies to SARS-CoV-2 among Health Care Workers in Kenya
Source: Clin Infect Dis. Author manuscript; Available in PMC 2022 Jan 31. (PMC8135298; doi:10.1093/cid/ciab346)
Supplement: Supplementary file [file EMS123289-supplement-Supplementary_file.docx]

Seroprevalence of Antibodies to SARS-CoV-2 among Health Care Workers in Kenya

**Authors**

Anthony O. Etyang^1^, Ruth Lucinde^1§^,Henry Karanja^1§^, Catherine Kalu^1^, Daisy Mugo^1^, James Nyagwange^1^, John Gitonga^1^, James Tuju^1^, Perpetual Wanjiku^1^, Angela Karani^1^, Shadrack Mutua^1^,Hosea Maroko^2^, Eddy Nzomo^3^, Eric Maitha^4^, Evanson Kamuri^5^, Thuranira Kaugiria^5^, Justus Weru^5^, Lucy B. Ochola^6^ ,Nelson Kilimo^7^, Sande Charo^8^, Namdala Emukule^9^, Wycliffe Moracha^10^, David Mukabi^10^, Rosemary Okuku^10^, Monicah Ogutu^10^, Barrack Angujo^1^, Mark Otiende^1^, Christian Bottomley^11^, Edward Otieno^1^, Leonard Ndwiga^1^, Amek Nyaguara^1^, Shirine Voller^1, 11^, Charles Agoti^1^, David James Nokes^1^, Lynette Isabella Ochola-Oyier^1^, Rashid Aman^12^, Patrick Amoth^12^, Mercy Mwangangi^12^, Kadondi Kasera^12^, Wangari Ng’ang’a^13^, Ifedayo Adetifa^1,11^, E. Wangeci Kagucia^1^, Katherine Gallagher^1,11^, Sophie Uyoga^1^, Benjamin Tsofa^1^, Edwine Barasa^1^, Philip Bejon^1,14^, J. Anthony G. Scott^1,11 §^, Ambrose Agweyu^1 §^, George Warimwe^1,14 §^

**Author Affiliations**

^1^KEMRI-Wellcome Trust Research Programme, Kilifi, Kenya

^2^KEMRI Center for Infectious and Parasitic Diseases Control Research, Alupe, Kenya

^3^ Kilifi County Hospital, Kilifi, Kenya

^4^ Department of Health, Kilifi County, Kenya

^5^ Kenyatta National Hospital, Nairobi, Kenya

^6^ Alupe Sub-County Hospital, Busia, Kenya

^7^ Institute of Primary Research, Nairobi, Kenya

^8^ Kocholia Sub-County Hospital, Busia, Kenya

^9^ Busia County Referral Hospital, Busia, Kenya

^10^ Department of Health, Busia County, Busia, Kenya

^11^ Department of Infectious Diseases Epidemiology, London School of Hygiene and Tropical Medicine, UK

^12^ Ministry of Health, Government of Kenya, Nairobi, Kenya

^13^ Presidential Policy and Strategy Unit, The Presidency, Government of Kenya

^14^ Nuffield Department of Medicine, Oxford University, UK

^§^Equal contribution

**1. Data collection form**

**2. Stan code for Bayesian adjustment of prevalence estimates to account for test performance**

data {

int N;

int N_se;

int N_sp;

int y;

int x;

int z;

}

parameters {

real<lower=0,upper=1> p;

real<lower=0,upper=1> se;

real<lower=0,upper=1> sp;

}

transformed parameters {

real<lower=0,upper=1> p_obs;

p_obs = se * p + (1 - sp) * (1 - p);

}

model {

//priors

p ~ beta(1, 1);

se ~ beta(1, 1);

sp ~ beta(1, 1);

//likelihood

y ~ binomial(N, p_obs);

x ~ binomial(N_se, se);

z ~ binomial(N_sp, sp);

}

**Data:**

|  | KNH | KCH | Busia | Nurse | Doctor | Clinical Officer | Pharmacy | Support staff | Other |
| --- | --- | --- | --- | --- | --- | --- | --- | --- | --- |
| y | 75 | 23 | 37 | 29 | 27 | 9 | 5 | 25 | 28 |
| x | 166 | 166 | 166 | 166 | 166 | 166 | 166 | 166 | 166 |
| z | 901 | 901 | 901 | 901 | 901 | 901 | 901 | 901 | 901 |
| N | 183 | 200 | 301 | 152 | 85 | 79 | 19 | 117 | 162 |
| N_se | 179 | 179 | 179 | 179 | 179 | 179 | 179 | 179 | 179 |
| N_sp | 910 | 910 | 910 | 910 | 910 | 910 | 910 | 910 | 910 |

**Table S1:** **Multivariable analysis of factors associated with presence of antibodies to SARS-CoV-2 in Nairobi, Kilifi and Busia**

| Characteristic |  | Nairobi | |  | Kilifi | |  | Busia | |
| --- | --- | --- | --- | --- | --- | --- | --- | --- | --- |
| Sex |  | OR | *(95% CI)* |  | OR | *(95% CI)* |  | OR | *(95% CI)* |
| Female |  | 1.0 | *–* |  | 1.0 | *–* |  | 1.0 | *–* |
| Male |  | 1.11 | *(0.60-2.05)* |  | 0.69 | *(0.26-1.81)* |  | 1.54 | *(0.74-3.19)* |
| Age category^§^ |  | 0.96 | *(0.73-1.26)* |  | 0.82 | *(0.52-1.30)* |  | 0.98 | *(0.69-1.39)* |
| Work in Covid Unit^±^ |  | – | – |  | – | – |  | 0.49 | *(0.16-1.51)* |
| Cadre |  |  |  |  |  |  |  |  |  |
| Nurse |  | 1.0 | *–* |  | 1.0 | *–* |  | 1.0 | *–* |
| Doctor |  | 0.87 | *(0.38-1.98)* |  | 1.40 | *(0.17-6.41)* |  | 4.62 | *(0.84-25.3)* |
| Clinical Officer |  | 0.44 | *(0.04-4.60)* |  | 1.18 | *(0.29-4.89)* |  | 1.68 | *(0.34-8.37)* |
| Support staff |  | 1.35 | *(0.47-3.86)* |  | 1.25 | *(0.24-6.43)* |  | 2.72 | *(0.76-9.75)* |
| Lab & Pharmacy |  | 1.24 | *(0.37-4.14)* |  | 2.44 | *(0.46-12.96)* |  | 1.89 | *(0.50-7.11)* |
| Other |  | 0.60 | *(0.25-1.44)* |  | 1.58 | *(0.38-6.56)* |  | 1.65 | *(0.48-5.74)* |

^§^ Per decade increase in age

^±^  Only applied to HCWs in Busia. None of the HCWs in Nairobi and Kilifi worked in a Covid isolation unit

**Figure S1: Study locations**

**Figure S2: Study flow chart**
